# Garcixanthone E and Garcimangophenone C: New Metabolites from *Garcinia mangostana* and Their Cytotoxic and Alpha Amylase Inhibitory Potential

**DOI:** 10.3390/life12111875

**Published:** 2022-11-14

**Authors:** Gamal A. Mohamed, Sabrin R. M. Ibrahim

**Affiliations:** 1 Department of Natural Products and Alternative Medicine, Faculty of Pharmacy, King Abdulaziz University, Jeddah 21589, Saudi Arabia; 2 Preparatory Year Program, Department of Chemistry, Batterjee Medical College, Jeddah 21442, Saudi Arabia; 3 Department of Pharmacognosy, Faculty of Pharmacy, Assiut University, Assiut 71526, Egypt

**Keywords:** *Garcinia mangostana*, xanthone, garcimangophenone C, garcixanthone E, benzophenone rhamnoside, cytotoxic potential, α-amylase inhibition, healthcare

## Abstract

*Garcinia mangostana* (Clusiaceae) is a rich pool of metabolites with diversified bioactivities. A new xanthone, garcixanthone E (**1**), and a new benzophenone, rhamnoside, as well as garcimangophenone C (**9**) together with garcinone E (**2**), α-mangostin (**3**), γ-mangostin (**4**), garcinone C (**5**), garcixanthone C (**6**), gartanin (**7**), and 2,4,6,3′,5′-pentahydroxybenzophenone (**8**) were purified from *G. mangostana* EtOAc extract. Their structural verification was accomplished utilizing assorted spectral tools and relating to the literature. The in vitro cytotoxic potential versus MCF-7, A549, and HCT-116 cell lines demonstrated the moderate potential of **1** (IC_50_s 8.5, 5.4, and 5.7 µM, respectively) in comparison to doxorubicin (IC_50_s 0.18, 0.6 and 0.2 µM, respectively) using a sulforhodamine B (SRB) assay. Additionally, **1** and **9** had AAI (α-amylase inhibition) with IC_50_s 17.8 and 12.9 µM, respectively, compared to acarbose (IC_50_ 6.7 µM). Further, their AAI mechanisms were inspected utilizing molecular-docking evaluation by employing the crystal structure of the human α-amylase (PDB-ID: 5EOF). Compound **9** possessed a reasonable docking score of −7.746 kcal/mol compared with the native ligand **7JR** which had a docking score of −9.932 kcal/mol. These results could further provide new insight into the potential usage of *G. mangostana* as a functional food for regulating postprandial hyperglycemia via suppressing AA.

## 1. Introduction

*Garcinia mangostana* (Clusiaceae, formerly Guttiferae) is among the most prevailing tropical fruits in southeast Asian regions. It has been widely consumed due to its high nutritional benefits, sweet unique taste, and pleasant aroma [1,2]. This plant has been famed in Chinese/Ayurvedic remedies for hundreds of years for treating various ailments, including cystitis, eczema, dysentery, gonorrhea, hyperkeratosis, gleet, psoriasis, and menstrual disorders [1,3,4]. Further, *G*. *mangostana* is known to possess diverse bioactivities, as it has anti-inflammatory, antimicrobial, antitumor, antimycobacterial, antioxidant, photoprotective, antimalarial, antiviral, and antileptospiral capacities [5,6,7]. Our former investigations of *G*. *mangostana* disclosed the characterization of xanthones as main metabolites, in addition to flavonoids, benzophenones, and phenolics [7,8,9]. Xanthones are a class of metabolites that are widely reported from lichens, plants, and fungi [10,11]. They are commonly produced by Polygalaceae, Gentianaceae, Guttiferae, Clusiaceae, and Moraceae plants [10,11]. They have an oxygenated tricyclic ring structure with diverse attached functional groups, such as methoxy, phenolic OH, and a dihydrofuran ring. These metabolites displayed various biological properties, including cytotoxic, antidiabetic, antioxidant, antileishmanial, antimicrobial, antimalarial, antitumor, antiHIV, antiquorum sensing, antihypertensive, anti-inflammatory, and larvicidal. Additionally, benzophenones are classes of metabolites reported from fungi and plants. It was stated that nearly 77% of benzophenones are separated from Clusiaceae plants [12]. These metabolites displayed various bioactivities, including antiHIV, antifungal, antiviral, antioxidant, and antimicrobial [12].

Cancer is one of the major serious illnesses that has a high, unacceptable mortality rate and incidence [13]. In total, 19.3 million new cancer cases and ≈10 million deaths because of cancer worldwide were estimated in 2020 [14]. Breast cancer with 2.3 million new cases (11.7%) has transcended lung cancer (11.4%) as the most frequently pinpointed cancer, followed by colorectal, prostate, and stomach (10.0%, 7.3%, and 5.6%, respectively) cancers. On the other hand, lung cancer with 1.8 million deaths (18%) continued to be the dominant reason of cancer death, following colorectal (9.4%) and breast (6.9%) cancers [14].

So far, the majority of anticancer agents have failed to accomplish the expected results. Therefore, there is an intensive research reorientation towards the discovery of new chemopreventive agents from natural sources [15,16]. Many natural metabolites are known to have chemoprotective potential towards various types of cancers worldwide [15]. These metabolites are widely found in fruits, vegetables, and plants [17]. It is a fact that consuming vegetables and fruits lowers carcinogenesis incidence [16]. Fruits and vegetables contain vitamins, fiber, minerals, and various bioactive metabolites, such as flavonoids, carotenoids, sterols, and phenolics, and all of them could be responsible for this protective potential [18].

The objective of this work was to discover new AAIs (α-amylase inhibitors) and anticancer agents from *G*. *mangostana* pericarps.

## 2. Material and Methods

### 2.1. General Experimental Procedures

A UV spectrum was accomplished utilizing a Hitachi-300 spectrometer (Hitachi High-Technologies Corporation/Kyoto/Japan). An ESIMS was performed with a LCQ-DECA mass spectrometer (Thermo_Finnigan/Bremen/Germany). An HRESIMS was executed utilizing a Micromass_Qtof2 spectrometer (Bruker/Rheinstetten/Germany). NMR spectra were determined on BRUKER_AVANCE600 equipment (BioSpin-Bruker/Billerica/MA/USA). IR data were estimated with an Infrared-400 Shimadzu spectrophotometer (Shimadzu/Kyoto/Japan). A chromatographic investigation was carried out on SiO_2_ 60 (0.04–0.063 mm)/Sephadex LH-20 (0.25–0.1 mm)/RP-18 (0.04–0.063 mm) (Merck/Darmstadt/Germany). Precoated SiO_2_60_F_254_ TLC plates (0.2 mm, Merck/Darmstadt/Germany) were employed for TLC examination. The metabolites’ purification and detection were carried out by employing a LiChrolut_RP-18 6 mL solid-phase extraction tube and UV inspection at λ*_max_* 366 and 255 nm and then spraying with H_2_SO_4_: *p*-anisaldehyde and a 110 °C heating.

### 2.2. Plant Material

*G*. *mangostana* fruits were secured in December 2019 from a Saudi local market. Its attestation was accomplished as earlier stated [6] and a voucher specimen (no. GM_1424) was kept in the herbarium at the Faculty of Pharmacy, KAU.

### 2.3. Extraction and Isolation

At room temperature, the dried pericarps (520 g) were extracted with MeOH (3 L × 5) until exhausting [7]. The combined concentrated methanol extract (GMT, 24 g) was suspended in distilled H_2_O (150 mL) and partitioned among *n*-hexane/EtOAc (500 mL × 6, each) to afford 2.7, 6.5, and 12.9 g, respectively, of *n*-hexane, EtOAc, and aqueous fractions. The EtOAC (6.5 g) fraction was chromatographed on SiO_2_CC (silica gel column chromatography) (300 g × 100 × 5 cm, EtOAC/*n*-hexane 5/95–0/100) to obtain four main subfractions: GME-1 (25/75), GME-2 (50/50), GME-3 (75/25), and GME-4 (100%EtOAc). The subfraction GME-2 (1.39 g) SiO_2_ CC (150 g × 50 × 3 cm, EtOAc/*n*-hexane gradient) produced 5 fractions of GME-2A: GME-2E. GME-2A (174 mg) SiO_2_ CC (30 g × 50 × 2 cm), EtOAC/*n*-hexane (10/90–30/70) provided **1**, which was purified on a RP-18 LiChrolut extraction tube (acetonitrile/H_2_O70/30– 20/80) to give a light yellow powder of **1** (9.6 mg). The GME-2B (295 mg) fraction was handled as GME-2A to result in **2** (22.4 mg). Additionally, GME-2C (478 mg) was managed as GME-2B to yield **3** and **4**, and their RP-18 column (100 g, 50 × 3 cm, H_2_O/MeOH gradient) produced **3** (29.4 mg) and **4** (13.7 mg). GME-3 (1.92 g) SiO_2_ CC (150 g × 50 × 3 cm, MeOH/CHCl_3_ gradient) resulted in 7 fractions: GME3A–GME3G. GME3B (320 mg) was handled on SiO_2_ CC (40 g, 50 × 2 cm, MeOH/CHCl_3_ gradient) following this RP-18 LiChrolut extraction tube (acetonitrile/H_2_O: gradient) and resulted in **5** (12.6 mg). The GME3C–GME3E (746 mg) fractions were combined relying on TLC and were submitted to Sephadex LH-20 (50 g, 50 × 3 cm, MeOH) to produce **6**, **7**, and **8**, and their further purifying on RP-18 (100 g, 100 × 3 cm, H_2_O/MeOH gradient) yielded **6** (9.2 mg), **7** (11.6 mg), and **8** (14.8 mg). The Sephadex LH-20 of GME-4 (100% EtOAc, 1.17 g) employing MeOH produced **9**, which was handled on an RP-18 column (H_2_O/MeOH (6:4–3:7) to obtain **9** (7.4 mg).

#### Spectral Data

Garcixanthone E (**1**)

Light-yellow powder. IR ν*_max_* (KBr): 2942, 3439, 1648, 1585, 1458 cm^−1^; UV (*λ_max_*, MeOH) (log *ε*): 237 (4.36), 269 (4.23), 322 (3.89), 386 (3.45) nm; HRESIMS *m*/*z*: 441.1908 (calc. for 441.1913, C_25_H_29_O_7_ [M+H]^+^); NMR spectral data (Table 1).

Garcimangophenone C (**9**)

Brown powder. IR ν*_max_* (KBr): 3365, 2985, 1638, and 1605 cm^−1^; UV (*λ_max_*, MeOH) (log *ε*): 310 (3.96), 282 (4.15), 213 (4.49) nm; HRESIMS *m*/*z*: 393.1180 (calcd for 393.1186 for C_19_H_21_O_9_ [M+H]^+^); NMR spectral data (Table 1).

### 2.4. In Vitro Cytotoxic Assay

The new compounds (**1** and **9**) were examined for cytotoxic potential towards human MCF-7 (breast cancer), HCT-116 (colorectal carcinoma), and A549 (lung cancer) cell lines using a sulforhodamine B assay (SRB) as previously stated [8].

### 2.5. a-Amylase Inhibitory Assay

The AAI potential of **1** and **9** was assessed utilizing Enz-Chek^®^ Ultra-Amylase Assay Kits as formerly stated [7].

### 2.6. Molecular Docking Evaluation

#### 2.6.1. Protein Preparation

To perform the docking studies, the crystal structure of the alpha amylase (PDB ID: 5E0F) was imported from the available online protein databank. Before docking, the protein was prepared by employing the Schrödinger suite protein preparation wizard tool [19]. The hydrogen and heavy atoms were subjected to optimization by restrained minimization. Additionally, missed H atoms were added, and the correct charges were assigned using the OPSL4 force field. H_2_O molecules from HET groups beyond 5 Å were removed.

#### 2.6.2. Ligand Preparation

Lig Prep was used to convert the compounds from 2D to 3D structures [20]. Strained minimization was carried out by employing the OPLS4 force field, the optimization of H-bonds was accomplished at pH 7.0 utilizing PROPKA, and water molecules beyond 3 Å were removed from the HET groups, Additionally, at 7.0 ± 2.0 pH, the metals’ HET cofactors and states were generated.

#### 2.6.3. Receptor Grid Generation and Docking

By using Glide, both ligands docking and grid generation were accomplished [21]. The grid box was defined by selecting the cocrystalized peptide inhibitor of 5E0F, and the binding region was specified using Glide’s Receptor-Grid-Generation tool. The generated grid was utilized for the prepared ligands docking using Glide software. The selected protocol was SP (standard precision). The default 0.25 potential charge cutoff and 1.0 radii scaling factor (vdW) were set. Compounds **1** and **9**, in addition to the cocrystalized ligand, 5,7-dihydroxy-4-oxo-2-(3,4,5-trihydroxyphenyl)-4H-chromen-3-yl-6-deoxy-2-O-{6-O-[(2E)-3-(3,4-dihydroxyphenyl)prop-2-enoyl]-beta-D-glucopyranosyl}-alpha-L-mannopyranoside (code:5JZ) and acarbose were redocked using the XP (extraprecision) protocol. All other settings were retained as the default.

## 3. Results and Discussion

### 3.1. Metabolites Purification and Structural Determination of ***1*** and ***9***

**Figure 1:**
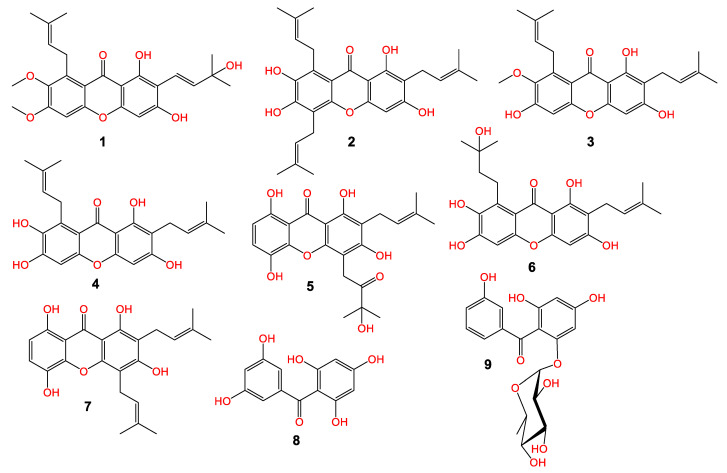
Chemical structure of new metabolites: garcixanthone E (**1**) and garcimangophenone C (**9**) and known (**2**–**5** and **6**–**8**) metabolites.

The MeOH extract of pericarps was partitioned among EtOAc and *n*-hexane. The EtOAC fraction was chromatographed utilizing SiO_2_ and CC to afford a new xanthone; garcixanthone E (**1**) and a new benzophenone rhamnoside; garcimangophenone C (**9**), along with garcinone E (**2**) [22], α-mangostin (**3**) [23], γ-mangostin (**4**) [24], garcinone C (**5**) [22], garcixanthone C (**6**) [9], gartanin (**7**) [25], and 2,4,6,3′,5′-pentahydroxybenzophenone (**8**) [26] (Figure 1). The former metabolites’ identification was achieved by comparing their spectral data to the earlier published ones and was proven via coTLC along authentic samples.

**Figure 2:**
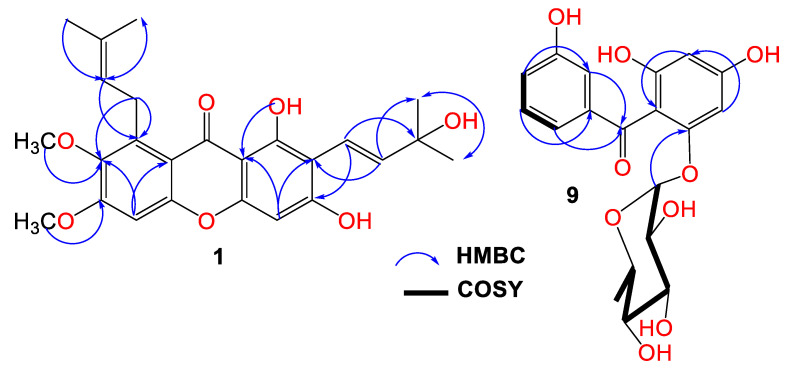
Important HMBC crosspeaks of **1** and **9**.

Compound **1** was purified as light-yellow powder with a C_25_H_28_O_7_ molecular formula, relying on the observed HRESIMS pseudomolecular peak at *m*/*z* 441.1908 [M+H]^+^ (calc. for 441.1913). This formula revealed 12 unsaturation degrees. The IR bands at 2942, 3439, 1648, 1458, and 1585 cm^−1^ characterized the presence of C-H aliphatic, OH phenolic, chelated carbonyl, C=C aromatic, and C–O functionalities, respectively [4]. It displayed UV absorptions for an oxygenated xanthone at 237, 269, 322, and 386 nm [9]. The HSQC and ^13^C exhibited 25 carbons: five methines, one methylene, six methyls, and twelve quaternary carbons, including an oxygen-linked aliphatic (δ_C_ 77.7, C-3′), five oxygenated-aromatic, and a carbonyl (δ_C_ 182.2, C-9) carbons (Figures S1–S4). The ^1^H revealed two singlets at δ_H_ 6.23 (H-4) and 6.88 (H-5) for two pentasubstituted phenyl moieties (Table 1). These signals related the carbons at δ_C_ 94.2 and 97.7 in the HSQC. The HMBC peaks of H-4/C-8b, C-2, C-3, and C-4a and H-5/C-8a, C-6, C-7, and C-4b affirmed these moieties. Additionally, a signal at δ_H_ 13.71 for chelated phenolic OH was observed. Its locality at C-1 was secured by C-1 (δ_C_ 157.9) and C-8b (δ_C_ 103.5) HMBC crosspeaks. The ^1^H and ^13^C spectra possessed disubstituted double bond signals at δ_H_ 5.55 (H-2′)/127.1 (C-2′), 6.72 (H-1′)/δ_C_ 115.8 (C-1′), two methyls at δ_H_ 1.46 (H-5′, 4′)/δ_C_ 28.5 (C-5′, 4′), and an oxygenated quaternary at δ_C_ 77.7 (C-3′), characterizing a 3-hydroxy-3-methylbut-1-enyl subunit. This was assured by the HMBC relations of H-1′/C-4′/C-2′/C-3′/C-5′, H-2′/C-4′/C-3′/C-5′, H-4′/C-2′/C-5′/C-3′, and H-5′/C-2′/C-4′/C-3′ (Figure 2).

Its connectivity at C-2 was established on the basis of H-1′/C-2/C-3, H-2′/C-1/C-2 HMBC crosspeaks. Further, the presence of the 3-methylbut-2-enyl subunit was evidenced by the noticed signals at δ_H_ 4.07 (H-1″)/δ_C_ 26.5 (C-1″), 5.25 (H-2″)/123.1 (C-2″), 132.2 (C-3″), 1.68 (H-4″)/25.6 (C-4′), and 1.82 (H-5″)/18.1 (C-5″). The H-2″/C-8 and H-1″/C-8/C-7/C-8a crosspeaks in the HMBC secured the location of the 3-methylbut-2-enyl subunit at C-8. Two methoxys at δ_H_ 3.75/δ_C_ 61.5 and δ_H_ 3.81/δ_C_ 59.9 were present. Their placements at C-7 and C-6 were asserted by the HMBC crosspeaks of 7-OCH_3_/C-7 (δ_C_ 142.6) and 6-OCH_3_/C-6 (δ_C_ 151.9). Therefore, **1** was designated as garcixanthone E (1,3-dihyroxy-6,7-dimethoxy-2-(3-hydroxy-3-methylbut-1-enyl)-8-(3-methylbut-2-enyl)-xanthone).

Compound **9** was obtained as a brown powder. Its IR spectrum revealed bands at 3365, 1638, and 1605 cm^−1^, which signalized the existence of hydroxyl, carbonyl, and C=C functionalities in **9**. Additionally, it had UV bands at 310, 282, and 213 nm [7,8]. The HRESIMS demonstrated a pseudomolecular peak at *m*/*z* 393.1180 (calcd for 393.1186 for C_19_H_21_O_9_), corresponding to the molecular formula C_19_H_20_O_9_, which required 10 unsaturation degrees for two benzene, one carbonyl, and hexose moieties. Further, the HRESIMS 246.0535 [M+H-hexose moiety]^+^ fragment peak indicated that **9** possessed a hexose sugar. In the HSQC and ^13^C, nineteen carbons were noticed, consisting of one methyl, eleven methines, and seven quaternary carbons for the oxygen-bonded aromatics at δ_C_ 163.8 (C4), 161.7 (C2), 159.5 (C6), and 158.2 (C3′), as well as one carbonyl (δ_C_ 197.5, C7) carbon. The NMR spectrum revealed signals at 6.95 (H-4′), 7.11 (H-2′), 7.17 (H-6′), and 7.19 (H-5′), having HSQC crosspeaks with carbons at 120.4, 116.9, 121.4, and 130.2, respectively (Figures S5–S8). The ^1^H-^1^H COSY relations of H-4′/H-2′ and H-6′, H-2/H-4′, H-6′/H-2′, and H-4′ featured the existence of a disubstituted benzene ring (substructure A). This was ensured by the observed HMBC crosspeaks of H-2′/C-1′/C-3′/C-4′/C-6′, H-4′/C-4′/C-2′/C-6′, and H-5′ and H-6′/C-1′/C-2′/C-3′/C-4′. In the HSQC, the carbons at δ_C_ 98.1 and 95.7 correlated to the metacoupled protons at δ_H_ 6.06 (H-3) and 6.21 (H-5), respectively, characterizing a substituted phloroglucinol moiety (substructure B). The HMBC crosspeaks of H-3/C-1/C-2/C-4/C-5 and H-5/C-1/C-2/C-4/C-3/C-6 emphasized this assignment. The connection between the two substructures through the carbonyl carbon was proved utilizing HMBC relations of H-5, H-3, H-2′, and H-6′/C-7 (δ_C_ 197.5). The ^1^H and ^13^C signals at δ_H_ 5.36 (d, *J* = 1.2 Hz, H-1″)/103.7 (C-1″) and δ_H_ 1.20 (H-6″)/δ_C_ 17.4 (C-6″), in addition to the signals at δ_H_ 3.82 (H-2′′)/72.4 (C-2″), 3.30 (H-3″)/72.6 (C-3″), 3.26 (H-4″)/73.6 (C-4″), and 3.69 (H-5″)/71.0 (C-2″) characterized a rhamnose moiety in **9**, which was ensured by the COSY and HMBC correlations [27]. Its attachment at C-6 was confirmed by the HMBC crosspeak of H-1″ to C-6 (δ_C_ 159.5). Based on these data, **9** was specified and named garcimangophenone C. It is noteworthy that this was the first report of isolating benzophenone rhamnosides from *G*. *mangostana.*

### 3.2. Cytotoxic and AAI (Alpha-Amylase Inhibitory) Activities

The cytotoxic potential of **1** and **9** was assessed towards MCF-7, A549, and HCT-116 cell lines using a sulforhodamine B (SRB) assay. Compound **1** had moderate activity towards A549, MCF-7, and HCT-116 with IC_50_s 5.4, 8.5, and 5.7 µM, respectively, compared with doxorubicin (IC_50_s 0.18, 0.6, and 0.2 µM, respectively). Unfortunately, **9** had weak cytotoxic potential versus the tested cancer cell line. It is mentionable that GM pericarp extracts revealed a significant glucose-decreasing and insulin-sensitization capacity [28]. It also revealed the antihyperglycemic effectiveness through boosting insulin-forming β-cell populations, which referred to its antioxidative phenolic constituents [29]. Moreover, it amended β-cells and pancreatic glands impairment caused by STZ in diabetic mice via promoting insulin production and modulating the sensitivity to the decreased insulin [30]. The treatments of diabetic mice with GM xanthones remarkably amended the antioxidant and biochemical parameters, reformed the kidney and liver histological changes, and lessened the kidney tissue cellular apoptosis [31]. Further, GM xanthones and benzophenones were proved to display α-amylase and α-glucosidase inhibitory capacities; therefore, they could minimize postprandial hyperglycemia via the prohibition of glucose absorption [7].

Accordingly, the new metabolites **1** and **9** were assessed for their AAI capacity. They demonstrated AAI potential (IC_50_ 17.8 and 12.9 µM, respectively) in comparison to acarbose (IC_50_ 6.7 µM).

### 3.3. Molecular Docking Evaluation

#### 3.3.1. Ligands and Protein Preparation

Compounds **1**, **9**, and **5J7** (native inhibitor of 5EOF) were prepared using LigPrep to convert 2D structures into 3D; additionally, the ionization state at a pH of 7.0 ± 2.0 and tautomeric forms were created. Using the protein preparation wizard, the human α-amylase’s protein crystal structure (PDB ID: 5E0F) was prepared, whereby the hydrogens were added, the bond orders were specified, and the het states using an Epik at pH 7.0 ± 2.0 were generated. The H-bonds were optimized at pH 7.0 employing PROPKA in sample water orientation, and the restrained minimization was performed using the OPLS4 force field.

#### 3.3.2. Receptor Grid Generation and Molecular Docking Studies

The grid box was created all over the protein’s binding site of the minimized protein that contained the cocrystalized inhibitor utilizing the crystal structure (PDB-ID: 5E0F), and the binding area was specified by the **5J7** native inhibitor’s selection. The nonpolar atoms were located and the Van der Waals radii scaling factor was set to 1, and 0.25 was the partial charge cutoff. The ligands docking was executed utilizing the Schrödinger suite “ligand docking” tool, the protocol was SP (standard precision), and all other settings were retained in their default form. The redocking of the ligand **5J7** (5,7-dihydroxy-4-oxo-2-(3,4,5-trihydroxyphenyl)-4H-chromen-3-yl 6-deoxy-2-O-{6-O-[(2E)-3-(3,4-dihydroxyphenyl)prop-2-enoyl]-beta-D-glucopyranosyl}-alpha-L-mannopyranoside) was performed to evaluate the docking study. Compound 9 exhibited a reasonable docking score (−7.746 kcal/mol) compared with the native inhibitor 5J7 (−9.932 kcal/mol) as shown in Table 2. For the docking validation, the native inhibitor was prepared and redocked alongside compounds **9** and **1**; then, the poses of the protein compound complexes were examined, and the RMS of the native inhibitor **5J7** was found to be in an acceptable range (1.0862).

**Figure 3:**
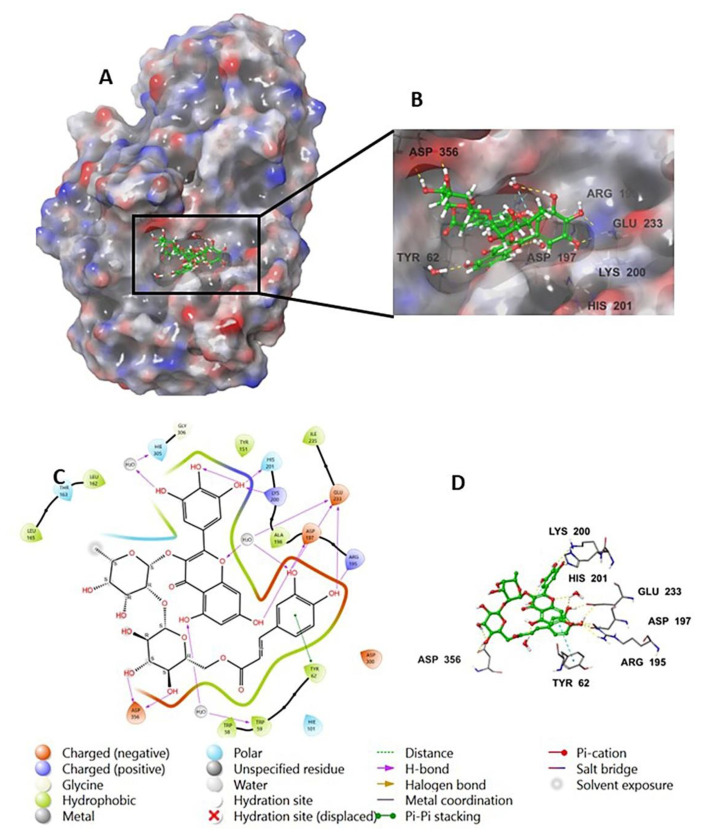
Native inhibitor (**5JZ**) in complex with human alpha amylase PDB: 5E0F; (**A**) molecular surface representation with solid style and electrostatic potential color scheme (red, white, blue) (min −0.3, max +0.3); (**B**) close lock to the human AA binding site with **5JZ**; (**C**) 2D representation of the binding interaction showing the important amino acids’ residues implicated in the interactions within 3 Å around the ligand; (**D**) 3D representation of the binding interaction, where the **5JZ** was represented in green color and wire representation was applied for amino acids’ residues; hydrogen bond represented in yellow dots and aromatic hydrogen bond represented in violet dots.

**Figure 4:**
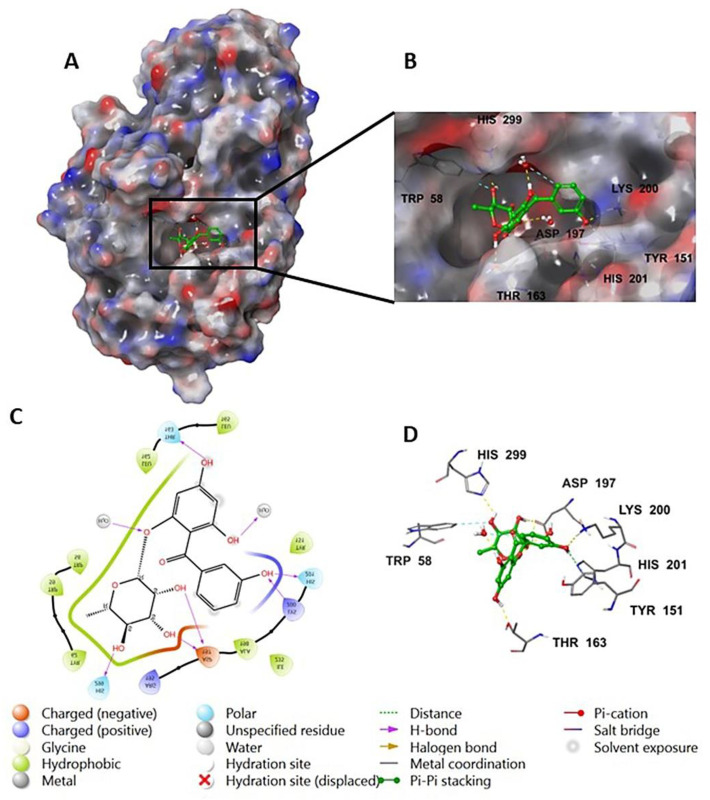
Compound **9** in complex with human alpha amylase (PDB: 5E0F); (**A**) molecular surface representation with solid style and electrostatic potential color scheme (red, white, blue) (min −0.3, max +0.3); (**B**) close lock to the human AA binding site with compound **9**; (**C**) 2D representation of the binding interaction showing the important amino acids’ residues implicated in the interactions within 3 Å around the ligand; (**D**) 3D representation of the binding interaction, where compound 9 was represented in green color and wire representation was applied for amino acids’ residues; hydrogen bond represented in yellow dots and aromatic hydrogen bond represented in violet dots.

The investigation of the cocrystalized human AA with the native inhibitor **5J7** showed the formation of hydrogen bonds and hydrophobic interactions with many amino acids’ residues. Hydrogen bonds formed between **5J7** and Gln63, Asp97, Glu233, and His305 while forming a hydrophobic interaction via pi-pi stacking with the residues Tyr62 and His 29 (Figure 3). Compound **9** interacted with the AA through hydrogen bonds between its hydroxyl groups and the amino acids’ residues Thr163, Asp197, Lys200, His 201, and His 299 in addition to the aromatic hydrogen bonds’ hydroxyl groups and TRP 59 and Tyr151 (Figure 4).

## 4. Conclusions

*G. mangostana* is one of the most valuable tropical fruits and its usage as a functional product has been growing because of its bioactivities that are related to its xanthones’ content. In the current study, two new metabolites, garcixanthone E (**1**) and garcimangophenone C (**9**), along with seven known compounds were separated from *G. mangostana* EtOAc extract using different chromatographic tools. Their structures were assigned based on various spectral analyses, including UV, IR, MS, and NMR. Compound **1** displayed moderate in vitro cytotoxic potential versus MCF-7, A549, and HCT-116 cell lines in the SRB assay. Additionally, **1** and **9** possessed moderate AAI potential. In the molecular docking study, **9** revealed a reasonable docking score compared to the native ligand **7JR** that agreed with the in vitro activity findings. These results could further prove a possible usage of *G. mangostana* as a functional food for treating diabetes and cancer. Certainly, more future in vivo and mechanistic studies are required to validate the activity of these interesting metabolites.

Further, to overcome the hazardous impacts and disadvantages of the conventional extraction of such metabolites by organic solvents such as MeOH, extraction using ecofriendly green solvents such as supercritical fluids, biobased solvents, and liquified gases could be applied. These solvents possess beneficial characteristics, including ease of preparation, biocompatibility, custom tunability, high selectivity, and low cost and volatility [32].

## Figures and Tables

**Table 1:** NMR data of garcixanthone E (**1**) and garcimangophenone C (**9**) (600 and 125 MHz).

		1 *			9 **		
No.	δ_H_ (mult., *J* (Hz)	δ_C_ (mult.)	HMBC	No.	δ_H_ [mult., (Hz)]	δ_C_ (mult.)	HMBC
1		157.9 C		1	-	109.4 C	-
2		104.7 C		2	-	161.7 C	-
3		160.1 C		3	6.06 d (2.0)	98.1 CH	1, 2, 4, 5, 7
4	6.23 s	94.2 CH	2, 3, 4a, 8b	4	-	163.8 C	-
4a		156.7 C		5	6.21 d (2.0)	95.7 CH	1, 2, 4, 3, 6, 7
4b		154.7 C		6	-	159.5 C	-
5	6.88 s	97.7 CH	4b, 8a, 6, 7	7	-	197.5 C	-
6		151.9 C		1′	-	142.8 C	
7		142.6 C		2′	7.11 d (1.8)	116.9 CH	1′, 3′, 4′, 6′, 7
8		137.0 C		3′	-	158.2 C	-
8a		112.0 C		4′	6.95 dd (7.8, 1.8)	120.4 CH	2′, 3′, 6′
8b		103.5 C		5′	7.19 d (7.8)	130.2 CH	1′, 2′, 3′, 4′, 7
9		182.2 C		6′	7.17 dd (7.8, 1.8)	121.4 CH	1′, 2′, 3′, 4′, 7
1-OH	13.71 s	-	1, 8b	1″	5.36 d (1.2)	103.7 CH	6
6-OCH_3_	3.81 s	59.9 CH_3_	6	2″	3.82 m	72.4 CH	1″, 3″, 4″
7-OCH_3_	3.75 s	61.5 CH_3_	7	3″	3.30 m	72.6 CH	1″, 5″
1′	6.72 d (15.4)	115.8 CH	2, 3, 2′, 3′, 4′, 5′	4″	3.26 m	73.6 CH	2″, 6″
2′	5.55 d (15.4)	127.1 CH	1, 2, 3′, 4′, 5′	5″	3.69 m	71.0 CH	2″, 3″, 6″
3′	-	77.7 C	-	6″	1.20 d (6.8)	17.4 CH_3_	3″, 4″, 5″
4′	1.46 s	28.5 CH_3_	2′, 3′, 5′	-	-	-	-
5′	1.46 s	28.5 CH_3_	2′, 3′, 4′	-	-	-	-
1″	4.07 d (6.0)	26.5 CH_2_	7, 8, 8a, 2″, 3″	-	-	-	-
2″	5.25 tq (7.8, 1.8)	123.1 CH	8, 4″, 5″	-	-	-	-
3″	-	132.2 C	-	-	-	-	-
4″	1.68 s	25.6 CH_3_	2″, 3″	-	-	-	-
5″	1.82 s	18.1 CH_3_	2″, 3″	-	-	-	-

* data were measured in CDCl3; ** data were measured in CD_3_DO.

**Table 2:** Results of in silico screening against human AA (PDB: 5E0F).

Compound	Docking Score	Glide Gscore	Glide Emodel
**Native_5E0F (5J7)**	−9.932	−9.966	−149.432
**9**	−7.746	−7.916	−83.594
**1**	−5.204	−5.309	−58.601

## Data Availability

Not applicable.

## Supplementary Materials

The following supporting information can be downloaded at: https://www.mdpi.com/article/10.3390/life12111875/s1: Figure S1: ^1^H NMR spectrum of compound **1** (600 MHz, CDCl_3_); Figure S2: ^13^C NMR spectrum of compound **1** (150 MHz, CDCl_3_); Figure S3: HSQC spectrum of compound **1**; Figure S4: HMBC spectrum of compound **1**; Figure S5: ^1^H NMR spectrum of compound **9** (600 MHz, CD_3_DO); Figure S6: ^13^C NMR spectrum of compound **9** (150 MHz, CD_3_DO); Figure S7: HSQC spectrum of compound **9**; Figure S8: HMBC spectrum of compound **9**.
