# Supplementary material for: Garcixanthone E and Garcimangophenone C: New Metabolites from Garcinia mangostana and Their Cytotoxic and Alpha Amylase Inhibitory Potential
Source: Life (Basel). 2022 Nov 14;12(11):1875. doi: 10.3390/life12111875 (PMC9696494; doi:10.3390/life12111875)
Supplement: Supplementary file 1 [file life-12-01875-s001.zip › life-2000522-supplementary.pdf]

life

Supplementary materials

Garcixanthone E and Garcimangophenone C: New Metabolites from *Garcinia mangostana* and their Cytotoxic and Alpha amylase Inhibitory Potential

Gamal A Mohamed[1,*], Sabrin R.M. Ibrahim[2,3]

[1] Department of Natural Products and Alternative Medicine, Faculty of Pharmacy, King Abdulaziz University, Jeddah 21589, Saudi Arabia
[2] Preparatory Year Program, Department of Chemistry, Batterjee Medical College, Jeddah 21442, Saudi Arabia; sabrin.ibrahim@bmc.edu.sa
[3] Department of Pharmacognosy, Faculty of Pharmacy, Assiut University, Assiut 71526, Egypt

*Correspondence: gahussein@kau.edu.sa

Figure **S1:** ^{1}H NMR spectrum of compound **1** (600 MHz, $CDCl_3$).
Figure **S2:** ^{13}C NMR spectrum of compound **1** (150 MHz, $CDCl_3$).
Figure **S3:** HSQC spectrum of compound **1**.
Figure **S4:** HMBC spectrum of compound **1**.
Figure **S5:** ^{1}H NMR spectrum of compound **9** (600 MHz, CD_3DO).
Figure **S6:** ^{13}C NMR spectrum of compound **9** (150 MHz, CD_3DO).
Figure **S7:** HSQC spectrum of compound **9**.
Figure **S8:** HMBC spectrum of compound **9**.

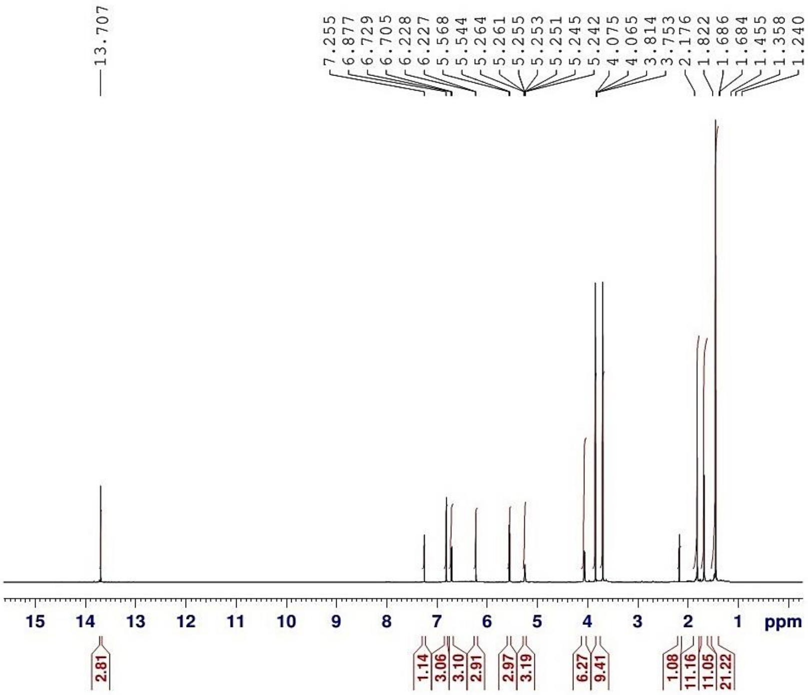

Figure **S1:** ^{1}H NMR spectrum of compound **1** (600 MHz, $CDCl_3$).

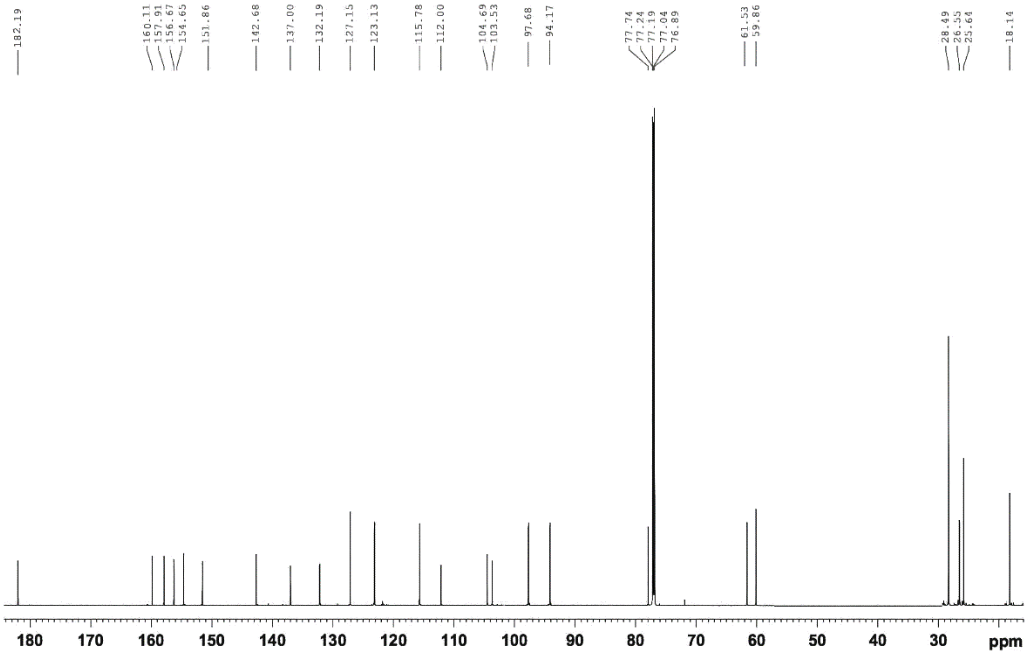

Figure **S2:** ^{13}C NMR spectrum of compound **1** (150 MHz, $CDCl_3$).

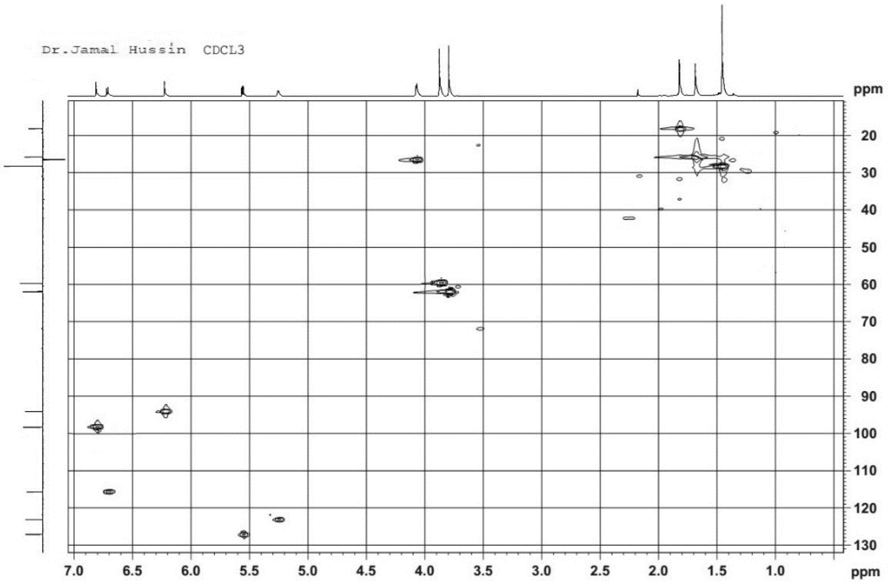

Figure **S3:** HSQC spectrum of compound **1**.

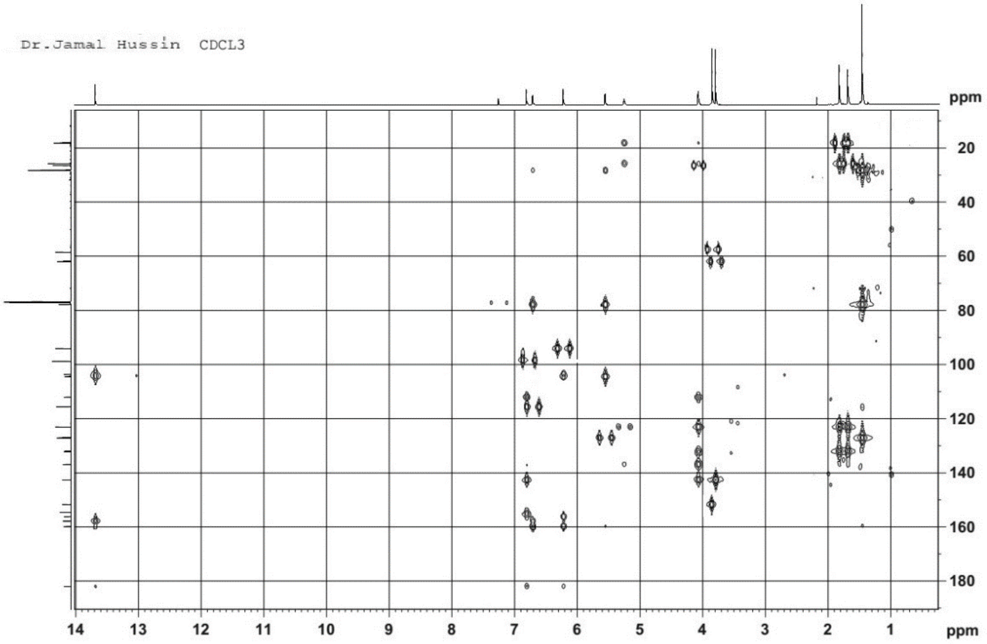

Figure **S4:** HMBC spectrum of compound **1**.

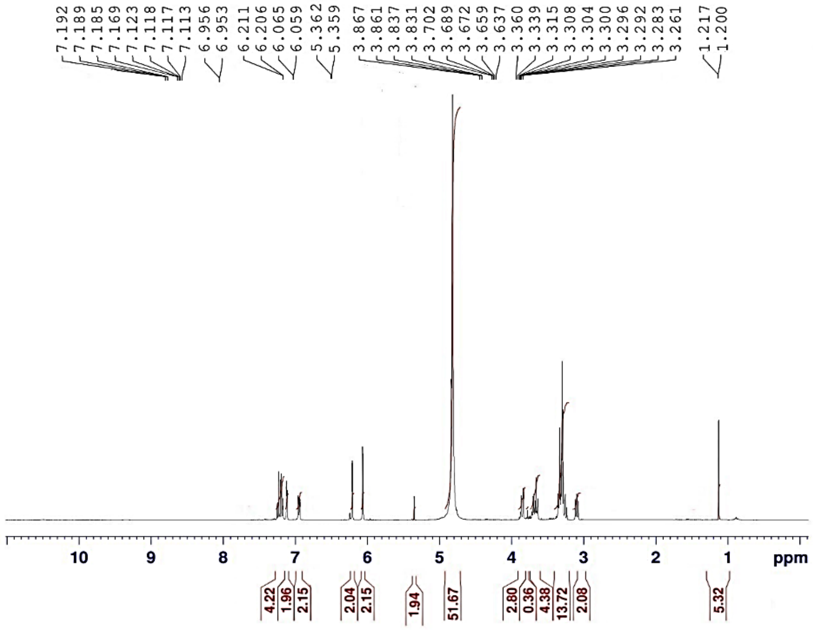

Figure **S5:** ^{1}H NMR spectrum of compound **9** (600 MHz, CD_3DO).

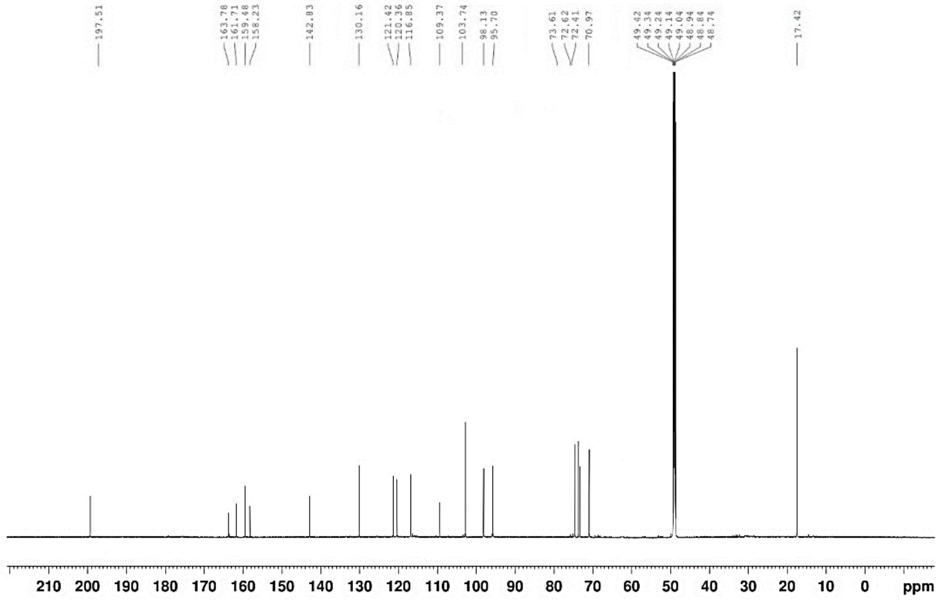

Figure **S6:** ^{13}C NMR spectrum of compound **9** (150 MHz, CD_3DO).

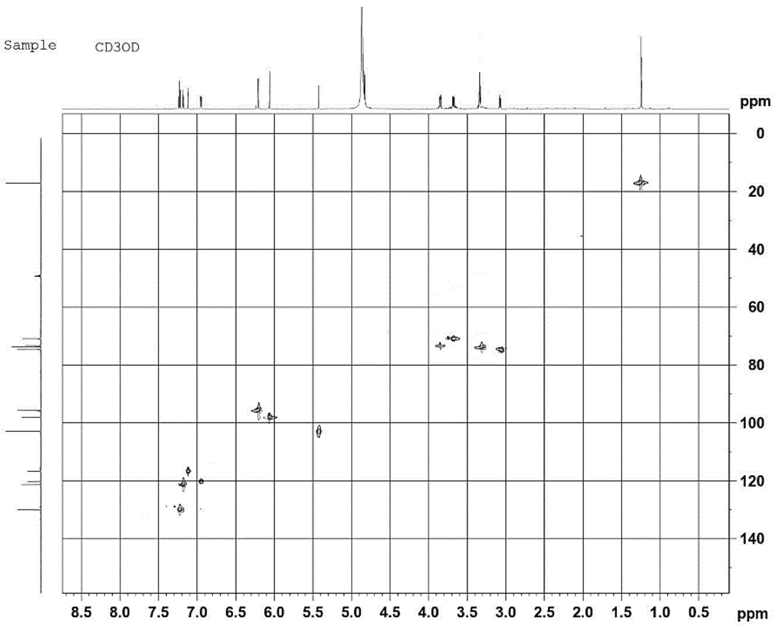

Figure **S7:** HSQC spectrum of compound **9**.

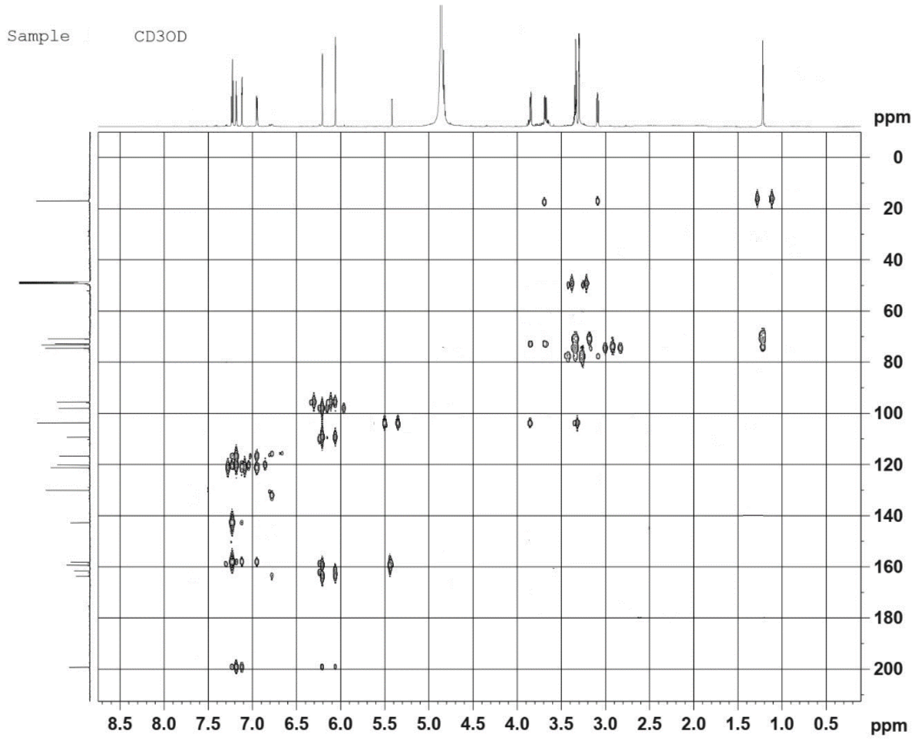

Figure **S8:** HMBC spectrum of compound **9**.
